# The validity and reliability study of the first-time fathers questionnaire in Turkish

**DOI:** 10.1016/j.heliyon.2023.e23957

**Published:** 2023-12-19

**Authors:** Ayça Demir Yildirim, Tuğba Yilmaz Esencan, Asa Premberg, Nevin Hotun Şahin

**Affiliations:** a Department of Midwifery, Faculty of Health Sciences, Uskudar University, Istanbul, Turkey; b Research and Development Centre, Primary Health Care, Gothenburg, Sweden; c Institute of Health and Care Sciences, Sahlgrenska Academy, University of Gothenburg, Gothenburg, Sweden; d Florence Nightingale Faculty of Nursing, Department of Women's Health and Diseases, Istanbul University-Cerrahpaşa, Istanbul, Turkey

**Keywords:** Fathers, Delivery-related experience, Reliability, Validity, Questionnaire, Antenatal education

## Abstract

The father's participation in the birth contributes significantly to the mother's birth experiences as well as to the fatherhood process. Since fathers have traditionally not been allowed to attend childbirth in Turkish public hospitals, which now is changing, there is a lack of research in this area. To remedy this shortcoming, a questionnaire that explores the Turkish fathers' experiences of childbirth is needed. The study aims to translate, validate, and adapt the pre-existing *First-Time Fathers' Questionnaire* to the Turkish language and culture. In the first stage of the study, the questionnaire was translated to Turkish. Expert opinions of the *First Time Father Questionnaire* were taken, and the content validity was checked. The revised questionnaire was then completed by 110 fathers. The average age of the fathers participating in the study was 32.12 ± 6.8 and 80 % of them were found to be middle class. The construct validity of the questionnaire was tested with explanatory factor analysis and confirmatory factor analysis, finally a test-retest was performed. The Cronbach's alpha coefficient for each dimension of the 20-items questionnaire was as follows: knowledge = 0.90, acceptance = 0.90, anxiety = 0.88, and emotional support = 0.66. All sub-dimensions correspond to 68.5 % of the total variance. The confirmatory factor analysis model showed consistency for the data (X2/DF = 1.078; RMSA = 0.027; CFI = 0.992; GFI = 0.900; AGFI.0.829). Therefore, the adaptation of the *First Time Fathers Questionnaire* is a valid and reliable instrument in evaluating the childbirth experiences of first-time fathers in Turkish culture.

## HİGHLİGHT

• The presence of fathers in delivery rooms is beneficial for expectant mothers.
• Being a father for the first time is a crucial life event.
• The fathers' presence during the birth has a positive effect on mothers and babies.
• This translation and evaluation of First Time Fathers Questionnaire (FTFQ)was found to be a suitable measurement tool for fathers' experiences during childbirth in Turkish culture.

## 1 Introduction

Childbirth is an important experience in which parents gain a new role in their lives. Professional assistance during labour can help women feel in control and able to deal with the pain of labour, which can help avoid unwanted childbirth experiences [1]. A family member, friend, midwife, or partner can offer company and support throughout labour [2]. The approach of labour companionship was introduced to Türkiye over the last three decades [1]. Labour companionship by fathers has become an increasingly common practice in resource-rich regions [3]. A growing body of research indicates that fathers are crucial to the childbirth process and that having them there throughout labour, being in the delivery room, is advantageous for everyone involved—the mother, the baby, and the father himself [[1], [2], [3], [4]]. In its latest recommendations on health promotion interventions for maternal and newborn health, the World Health Organization strongly recommends promoting men's involvement during pregnancy, childbirth, and after birth [5].

The literature shows that fathers can receive limited attention from midwives and that they are not always involved during labour and childbirth. Fathers who are less involved can have a more negative birth experience. When fathers feel left out, this can be associated with panic and helplessness. A negative birth experience can also lead to symptoms of post-traumatic stress disorder [6]. Although there are numerous advantages to having fathers present through labour, recent research has shown that some men may also feel negative emotions, such as worry and anxiety, and may feel put aside by the healthcare staff when their partners are giving birth [2]. For example, according to research, it has been found that a negative birth experience may result in lower parental well-being and higher (parental) stress symptoms in partners [7]. Added to this, fathers were commonly not allowed to talk about their concerns, thus leaving these concerns unresolved and which would negatively influence the parent-child relationship [8]. Although the fathers' companionship in childbirth has increased worldwide in the last 50 years, this situation is still not common in Türkiye [9]. The reasons are gender-based traditions, the domestic roles of mothers and fathers, cultural factors, the national health system, a lack of policies and practices that support fathers' participation in childbirth, the roles of healthcare professionals during childbirth, and the fathers' insufficient knowledge about prenatal education [10]. A recent study conducted by Tastan and Duru [1], in Türkiye found that the percentage of fathers’ willingness to participate in delivery was 71.8 %.

Pregnant women need support and feel that they need to be considered important in order to cope with delivery and have a healthy birth process. Professional support provided during the labour can give these women feelings of control and the ability to cope with labour pain, and it can prevent negative experiences during delivery [7,11]. Support can be provided by the spouse, relatives or other family as well as midwives [12,13].

The presence of a woman's spouse during delivery helps her cope with pain and prevents her from losing her control. Thus, spouses' participation during delivery is considered to be beneficial and indispensable [14,15].

Being a father for the first time is a crucial life event [16]. Emotions such as excitement, joy, happiness, curiosity, anxiety, and fear are felt intensively during the delivery. Emotional support of partners in the delivery room is important [17]. The presence of fathers in the delivery room has resulted in benefits, and these benefits have become a significant milestone for the fatherhood role. This important role of the father has increased the scientific interest in fathers during childbirth. Since the experiences of fathers' companionship in childbirth in Türkiye are not known enough, there is insufficient data to plan care and education content for fathers concerning childbirth [11]. In order to eliminate these inadequacies, there is a need for each culture-specific instrument measuring the fathers' birth experiences. As far as we know, there is no measurement tool to evaluate these experiences in Turkish culture in the available literature.

When evaluating fathers' participation in childbirth, there are the following: City Birth Trauma Scale (Partner Version), Birth Participation Scale (BPS), and First-Time Father Questionnaire [16,18,19]. According to the literature, there are two questionnaires that have been developed to assess fathers’ experience of childbirth [[20], [21]]. The only one that examined the childbirth experiences of first-time fathers was the First-Time Father Questionnaire (FTFQ) [22]. Studies using the FTFQ stated that it is effective in evaluating the fathers' preparedness for childbirth. In addition, the FTFQ enables fathers to determine the support they received from the birth and their feelings during the birth [22,23]. The FTFQ, developed in Sweden, was specifically designed for first-time fathers [16], and has subsequently been translated into various languages (English, Spanish, French, and Chinese). All versions of the FTFQ have acceptable validity and reliability [2,24,25].

As there is no validated questionnaire to assess fathers’ experiences of childbirth in Turkish, this methodological study aimed to translate, adapt, then validate the FTFQ to see if it was applicable to the Turkish language and culture.

## 2 Materials and methods

### 2.1 Research model

For this study, a causal comparative model was used to develop the FTFQ Scale. The causal comparative model aims to identify the possible variables that constitute the dependent variable [26].

### 2.2 Setting and participants

This study took place in a prenatal education centre. The study population consisted of the husbands of pregnant women who received antenatal parenthood education at a public hospital in Türkiye. Informed consent was obtained from all participants. The data were collected by the researcher during face-to-face interviews conducted with each participant for approximately 20 min. The FTFQ was translated and adapted to the Turkish language following the methodological criteria. The FTFQ consists of 22 items and 110 fathers were included in the study by adjusting the number of fathers for the sample to be more than five times the number of items. All of the fathers that were included in the study answered the questionnaires [27].

The fathers in the sample were 92.7 % university graduates and the average age was 32.12 ± 6.8. Additionally, it was observed that 80 % of them were found to be middle class. Demographic information is provided in Table 1.

**Table 1:** Socio-demographic Characteristics of the study group.

Socio-Demographic Variables			
Age(n = 110) x̄±s = 32.12 ± 6.8			
		n	%
**Marital status (n = 110)**	Married/Lives with a partner	110	100
	Single	0	0
**Educational Status (n = 107)**	Primary School	1	0.9
	Secondary School/High School	4	3.6
	University	102	92.7
**Income Status**	Low	3	3.4
	Medium	90	81.2
	High	17	15.4
**Delivery Type (n = 110)**	Normal delivery	70	63.6
	Vacuum/Forceps	3	2.7
	Caesarean section	37	33.6
**Preparation to Delivery**^a^	Self-Preparation	28	25.5
	Preparation through Internet	44	40.0
	Gaining Information from Family and Friends	45	40.9
	Taking a Prenatal Course at a Private Institution	48	43.6
	Taking a Prenatal Course run by the Ministry of Health	47	42.7
	Gaining Information from the Doctor	13	11.8
	None	9	8.2
**Place of Delivery (n = 110)**	State Hospital	3	2.7
	Training and Research Hospital	3	2.7
	Private Hospital	97	88.2
	Private University Hospital	3	2.7
	Home	4	3.6
**Volunteering to Participate in Birth**^a^	Own request	70	63.5
	His partner's request	49	44.5
	Reluctant	10	9.1
	Deciding through education	14	12.7

a Multiple options were present.

Criteria for including the cases in the study.

- Father's attendance at antenatal parenthood sessions
- Accompanying the partner during labour
- Being a father for the first time
- Participation in delivery for the first time
- Ability to speak Turkish

### 2.3 Instrument, variables and measurement

Data were collected using the First-Time Fathers Questionnaire (FTFQ) developed by Premberg, Taft, Hellstrom & Berg [16]. The document assesses the delivery-related experiences of first-time fathers using four dimensions: worry (8 items), knowledge (4 items), emotional support (6 items) and acceptance (4 items). The FTFQ includes 33 items, of which 22 (items 5–26) assess first-time fathers’ experience of childbirth and the other 11 items collect background information on socio-demographic data. The mean score is calculated for each item of the four dimensions. These scores are included in the calculation on the condition that at least 50 % of the items constituting the sub-dimension have been answered. The instrument is of a 4-point Likert type, with a score range of 1–4 for each dimension, and higher scores indicate worse experiences. The total score may not be calculated.

The instrument was translated into Turkish by four different scientists who were experts in their fields (two gynecologists, a nurse specialized in women's health and diseases, and a specialist midwife). Similar translations of these experts were utilized for the selection of items, and these translated items were re-translated into English by an English-speaking expert. The Turkish version of the questionnaire was developed in collaboration with Premberg, Taft, Hellstrom & Berg [16], and it helped ensure a significant similarity between the Turkish version and the English translation of the questionnaire.

During the first step of the study, the thoughts of 15 experts (Obstetricians, gynaecologists, and midwife academics) were assessed concerning the Turkish version of the questionnaire. Each item of the questionnaire was assessed by the experts as follows: 1 = Not appropriate, 2 = The item should be revised appropriately, 3 = Appropriate but requires minor changes, and 4 = Quite appropriate. Every item was to be scored between 1 and 4. A Content Validity Index (CVI) assessment was used for the scores experts gave to the items. The questionnaire was validated by four people: a native English-speaking physician, a sworn translator, an obstetrician, and an academic midwife.

### 2.4 Test-retest

In the study, a second evaluation (test-retest) was made with 30 fathers. This was made three days after the first evaluation (test) so that they would not remember the questionnaire.

## 3 Statistical methods

### 3.1 Ethical Committee approval

Ethical Committee of Clinical Research (decision dated April 17, 2019/numbered 41) at Zeynep Kamil Maternal and Children Diseases Training and Research Hospital approved the study. All procedures performed in studies involving human participants were made following the ethical standards of the institutional and/or national research committee and with the 1964 Helsinki Declaration and its later amendments or comparable ethical standards. Written permission for the use of the questionnaire was obtained.

## 4 Results

This demographic information was chosen as it is important for the validation of the study and to give the possibility to compare with other studies. Linguistic validity, content validity and construct validity of FTFQ were analysed to test the validity of the questionnaire. Finally a test-retest was performed.

### 4.1 Linguistic validity

During the first stage of the study, the linguistic validity of the questionnaire was examined. First, the FTFQ was translated from English into Turkish by four scientists who were native in both languages and the items commonly translated to Turkish by these experts were re-translated into English by another expert who was a native English speaker. The experts made translations that were not very different from each other. The only translation differences were found concerning support services during childbirth, which were different from our health system, and cultural-specific translations were standardized.

### 4.2 Content validity

When the content validity of the FTFQ was determined, the original form of the questionnaire and its Turkish version, the linguistic validity of which had already been performed, were sent to 15 academics via email. The experts were asked to assess whether the items of the questionnaire, that had already undergone linguistic validity assessment, were understandable and suitable for the Turkish culture. Academics used the Davis Method to assess the items. Accordingly, each item was assessed by them as follows: 1 = Not appropriate, 2 = The item should be revised appropriately, 3 = Appropriate but requires minor changes, and 4 = Quite appropriate. Based on the experts’ recommendations, some items were revised. Item 28 of the questionnaire, "I was worried that I would not be able to provide support", was changed to "I was worried that my support would not be sufficient". The original sentence had been modified based on expert opinions when translated into English, as it did not have an exact equivalent in Turkish, in terms of meaning.

As the Davis Method (Scope Validity İndex calculation technique) was used, the item-related CVI was found, and the number of experts who selected options 3 and 4 was divided by the number of all experts who assessed the questionnaire. Davis proposes that researchers should consider 80% agreement or higher among judges for new research instruments [18]. Judgment on each item was made as follows: If the I-CVI is higher than 79%, the item will be appropriate. If it is between 70 and 79%, it needs revision. If it is less than 70%, it is eliminated. According to the literature, the CVI score should be over 0.80 [25]. All items in the questionnaire were revised according to expert recommendations. CVI calculations were performed for the items and only two items scored below 0.80, while all other items scored above 0.80. Since there were no items that fell below the threshold value according to the CVI calculation, no item was removed from the questionnaire.

### 4.3 Construct validity

The EFA and CFA were used for testing the construct validity of the questionnaire.

### 4.4 Explanatory factor analysis

The KMO test was performed to see whether the samples in the study sufficed for the factor analysis and Bartlett's Test of Sphericity was conducted for the practicability of the factor analysis. Table 2 presents the results of the KMO test and Bartlett's Test of Sphericity.

**Table 2:** Results of KMO test and Bartlett's test of Sphericity.

Tests	Results	p*
KMO coefficient	0.789	
Bartlett's Test of Sphericity	*X*^2^ = 1271.854	0.000

*p < 0.05.

The results of the tests indicated a KMO coefficient of 0.789. Accordingly, the volume of the sample was sufficient for the factor analysis. Furthermore, the results of Bartlett's Test of Sphericity indicated the chi-squared value to be p < 0.01. As this value was significant, it was understood that factor analysis could be performed on the sample.

The questionnaire was found to have four factors. The load factors of the questionnaire indicating distributions of items in one factor following the Varimax Rotation Method are presented in Table 3.

**Table 3:** Factor structure and loading of the Turkish FTFQ (20 items).

Item	Knowledge	Acceptance	Worry	Emotional Support
I5 I felt well informed.	.827			
I6 I felt well prepared.	.866			
I7 We were admitted to the maternity unit we selected.		.593		
I8 I was well received when I phoned the maternity ward.		.865		
I9 I was treated well when I came to the maternity unit.		.913		
I10 I felt I was given positive attention by the staff		.902		
I11 I was given enough information		.871		
I12 I received guidance from the health personnel in terms of how I could support my wife/partner.		.608		
I15 I felt worried about my wife/girlfriend.			.798	
I16 I was worried about the baby.			.846	
I17 I was worried, thinking that something might go wrong.			.830	
I18 I was worried that I wouldn't be able to provide support.			.793	
I19 I was worried about the uncertainties.			.788	
I20 I was worried about how I would react during the delivery.			.692	
I23 Things that scared me during labour happened			.550	
I21 I felt that midwives and other personnel cared about how I felt.				.692
I22 The health personnel offered to support my wife/partner when I wanted to take a break.				.692
I24 I received support from the health personnel when I was upset.				.624
I25 The health personnel showed me how to hold the baby.				.901
I26 I was encouraged by the health personnel to hold the baby in my arms				.897

The original questionnaire has 22 items. Following the item factor analysis, two items (Item 13, “There was an informational deficiency,” and Item 14, “There were certain things I preferred not to have experienced”) met the exclusion criteria (<0.20) and were greater than p > 0.05 after the *t*-test were excluded from the questionnaire. The final sub-dimensions corresponded to 68.5 % of the total variance with 20 items: *Worry, Knowledge, Emotional* Support *and Acceptance.*

Worry (seven items): Includes items related to concerns regarding the well-being of the partner and baby, insufficiencies in providing support, self-reactions and fears regarding uncertainties.

Knowledge (two items): Includes items related to being prepared during delivery and knowing about the delivery.

Emotional support ( five items): Includes items related to the fathers’ experiences of receiving guidance and support from the health personnel and being comfortable during the delivery.

Acceptance ( six items): Includes items related to how fathers were met, treated, and approved by the healthcare providers.

### 4.5 Reliability

The correlation of questionnaire items as the sub-dimensions of the questionnaire was examined and it was found to be over 0.40. Cronbach's Alpha coefficients regarding the sub-dimensions of the questionnaire were found to be acceptable for the group analyses (>0.77). Cronbach's Alpha coefficients regarding the sub-dimensions were as follows: Knowledge (0.90), Acceptance (0.90) and Worry (0.88). Despite these good figures, the coefficient of Emotional Support (0.66) was relatively lower (Table 4).

**Table 4:** FTFQ sub-dimensions' item–total score correlations.

Questionnaire	Item internal consistency				
	Item-Questionnaire Correlation	Cronbach's Alpha	Cumulative Variance	Correlation range	Variance range
**Worry**	7	0.880	58.75	0.30–0.72	1.10–1.39
**Knowledge**	2	0.905	91.33	082.082	0.66–0.71
**Emotional Support**	5	0.668	43.49	0.11–0.73	1.10–1.59
**Acceptance**	6	0.902	68.59	0.26–0.88	0.72–1.02

### 4.6 Confirmatory factor analysis (CFA)

Following the explanatory factor analysis, confirmatory factor analysis was used to understand how the questionnaire fitted the real data, and an assessment was made as to whether the 20-item structure of the questionnaire was confirmed. The fit index values found at the end of the confirmatory factor analysis are presented in Table 5.

**Table 5:** Confirmatory factor analysis.

Factors of Fitness	Good Fitness	Acceptable Fitness	Results of the Model
The Root Mean Square Error of Approximation (RMSEA)	0 < RMSEA <0.05	0.05 < RMSEA <0.08	0.025
Goodness of Fit Index (GFI)	0.95 < GFI <1	0.90 < GFI <0.95	0.901
Adjusted Goodness of Fit Index (AGFI)	0.90 < AGFI <1	0.80 < AGFI <0.90	0.830
Comparative Fit Index (CFI)	0.95 < CFI <1	0.90 < CFI <0.95	0.993
χ^2^/sd	χ^2^/sd < 2	2 < χ^2^/sd < 5	1.070

**Fig. 1:**
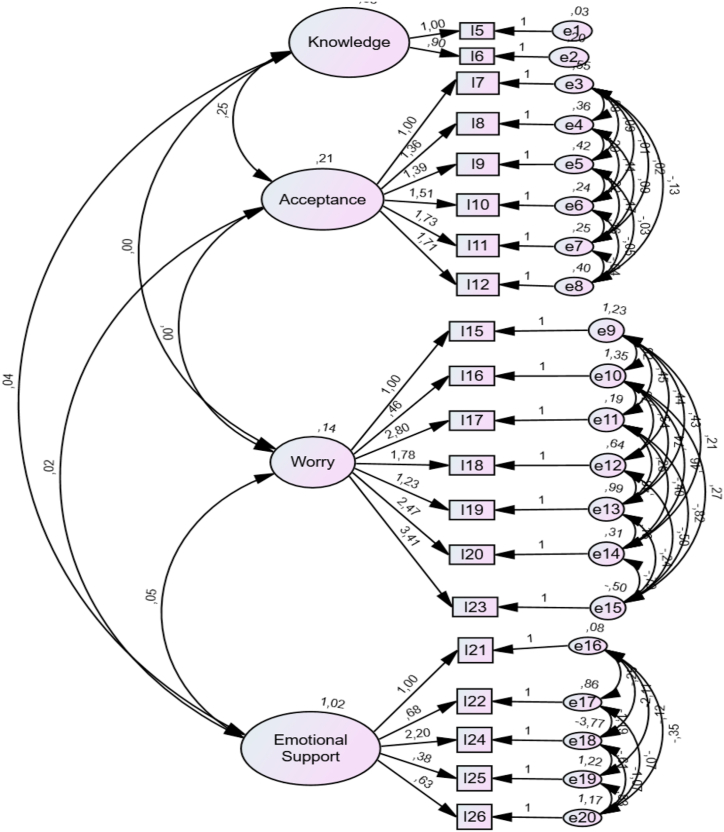
Factor loads between the factors and items.

The coefficients indicating the relationship between the variables and factors of the model showing the factorial structure of this questionnaire revealed that all coefficients were at a sufficient level. The fit index statistics calculated using the CFA indicated that the pre-determined structure of the questionnaire indicated fitness to the collected data at an acceptable level. By following the CFA, a path diagram regarding the factor loads between the factors and relevant items was formed and is presented in Fig. 1.

To find the group that created the difference, the Bonferroni test, which is a post hoc paired comparison test, was used. When the sub-dimensions of the questionnaire were compared with the form of delivery, the Knowledge, Acceptance and Emotional Support parameters were found to have no impact, whereas the Worry sub-dimension had a statistically significant relationship with the caesarean section (Table 6). Finally the test-retest evaluation, the correlation coefficient of the questions was found to be 0.652 (p = 0.004).

**Table 6:** Comparison of sub-dimensions and Form of delivery.

	Vaginal Delivery (a) (n = 70)	Interventional Delivery (b) (n = 3)	Caesarean Section (c) (n = 37)	Analysis of Variance p	Post hoc a–b	Post hoc a–c
Knowledge	3.31	4.00	3.67	0.450	1.00	0.80
Acceptance	9.27	13.0	9.83	0.336	0.48	0.72
Worry	19.47	12.66	16.51	0.012	0.14	0.41
Emotional Support	11.91	11.86	11.88	0.966	1.00	1.00

## 5 Discussion

This study assessed the FTFQ, which evaluates first-time fathers’ experience while participating in delivery and the questionnaire was tested for validity and reliability in Türkiye. The Turkish validity and reliability tests of the translated FTFQ were performed and 110 fathers who completed the FTFQ were included. The Turkish version of FTFQ presents a suitable factorial structure related to the original FTFQ. With care and attention the Turkish version of the questionnaire protected four factors from the original version. Relevant studies performed in other languages and cultures indicated that our study most resembled the Chinese version of the original FTFQ [2]. Three factors were preserved in the French version of the FTFQ [24], and two factors of the FTFQ that were assessed in Spanish, in Latin America, were conserved [25].

The cumulative variance contribution of the four factors in the study was 68.5 %, with the factor load of each item ranging from 0.55 to 0.91. The variance of four factors was 48 % in the original form of the questionnaire and the factor load of each item ranged from 0.41 to 0.82 [16]. The data of the present study had similar results to the Chinese version of the FTFQ in that the cumulative variance contribution of four factors was 64.65 %, and the factor load of each factor ranged from 0.56 to 0.89 [2]. In the instrument validity and reliability study conducted in French, the cumulative variance contribution of the four factors was 54.12 %, and the factor load of each factor ranged from 0.43 to 0.89 [24]. Compared to the results of studies in other languages, Turkish FTFQ had a better language performance than the French FTFQ in terms of construct validity [24]. The cumulative variance contributions of the Chinese and Turkish versions were close [2].

The original form of the questionnaire has 22 items [16]. Following the item factor analysis of the Turkish version, two items that met the exclusion criteria (<0.20) and had p > 0.05 after the *t-*test were excluded from the questionnaire. Of these two items, question 13, “There was an informational deficiency”, and question 14, “There were certain things I preferred not to have experienced,” did not meet the minimum criteria for the homogeneity of the study. These items cover the evaluation of the clinic. The discrepancies are thought to be caused by cultural differences.

Prenatal training sessions of pregnancy schools help in the preparation for delivery in Türkiye, so the couples' informational deficiencies are met, and contributions are made to the couples' preparation [21]. Significant changes have occurred in the fathers' participation in pregnancy and delivery and paternal roles in Türkiye; this is also valid for other countries [[21], [28], [29], [30]]. This current paternal role, called ideal, and modern fatherhood, requires fathers to connect with their children through strong ties: physical, emotional, economic, and social responsibilities. Fathers should share parental roles with the mother, and to take more responsibilities in terms of perinatal care, accompanying the partner during delivery, and providing new-born care [29,31,32]. In a qualitative study conducted regarding the fathers’ participation in delivery following prenatal training, the fathers contributed to the process of ensuring the security of mothers and babies, understood their support role by relieving labour pains, and providing postnatal care and a theme of communication [33].

In this study, there is a relationship between education level, childbirth preparation training, midwife support, and the fathers' willingness to participate in childbirth. In an experimental study conducted in Türkiye, the fathers' support helped the mothers experience a more positive birth [10]. Mothers gain more positive experiences when supported by their partners during pregnancy and delivery [30,33]. Furthermore, midwives have a great impact on improving the fathers' roles during delivery [30]. Midwives may provide culturally suitable and sensitive care to fathers during pregnancy and delivery, and they may contribute to improving the quality of fathers’ parental role and achieving more positive results for mothers and babies [29,34].

Compared to the original questionnaire, the internal consistency was well achieved [16]. Yielding similar results in terms of internal consistency for Cronbach's Alpha value, ranged from 0.72 to 0.86 for four catchment areas, as in the Chinese FTFQ version [2]. Furthermore, in the assessment of the model fitness in CFA, the χ^2^/df value indicates the probability of correctness with relevant fitness values; as the model got closer to 0, the data observed consisted proportionally (χ^2^/df < 2). The pre-determined structure of the Turkish FTFQ achieved acceptable fitness to the collected data. RMSEA is a fit index which assesses how distant the hypothesized model is from a perfect model [35]. The RMSEA value was expected to be between 0 and 1. As this value gets closer to 0, the real data reflects the model more closely. From a general perspective, RMSEA> 0.10 indicates a weak fit, whereas 0.08 <RMSEA <0.10 indicates a moderate fit, 0.05 <RMSEA <0.08 suggests a reasonable fit, and RMSEA <0.05 means a good fit [36,37]. The RMSEA value was 0.027 in the present study, indicating good fitness between the theoretical model and the data observed. The same value was 0.03 in the Chinese version, indicating a resemblance to the data of the present Turkish study [2].

Most of the studies in scientific literature have focused on the mothers' birth experiences rather than the fathers' birth experiences. In a study conducted in France and Switzerland, which was one of the studies conducted examining the birth experiences of first-time fathers, in terms of professional support, anxiety, and prenatal preparation, it was seen that the vast majority of the fathers did not receive childbirth preparation training. It was determined that fathers who received prenatal education had lower birth anxiety [24]. In the study conducted by Baldwin et al., it was stated that fathers experienced a lack of information and support during childbirth and this caused them to feel excluded from the health services [38]. It was seen that 90 % of fathers in the USA received information and advice, about childbirth preparation, from their spouses, parents, friends, and colleagues [30]. In a systematic review examining the experiences and needs of first-time fathers during childbirth, it has been revealed that men who will become fathers for the first time experience insecurity, anxiety, lack of knowledge, and they need to make preliminary preparations. First-time fathers need to be able to cope with both positive and negative situations. The fathers need to be supported with guidance, professionalism, and knowledge in addition honest answers are required. First-time fathers want to feel that they are involved; this can be achieved by involving them in communication about processes and procedures and decission making. In this study, it was stated that fathers, as found in previous research papers, had difficulty getting support [39]. These results show that it is important for fathers to attend birth preparation classes. For this reason, the fathers included in the sample in this study were selected from among the fathers who had participated in birth preparation training. Like other studies conducted with fathers, this study found a statistically significant relationship between the anxiety sub-dimension and caesarean delivery.

## 6 Strength and limitations

The study's limitations are that the tool has not been tested in a sample outside the one in which it was validated. Another limitation is that, to validate an instrument, it is usually preferred to have a larger sample i.e. 10 people per item to be tested which would be 10x20 = 200. In our country, since fathers were not admitted to maternity hospitals to support the mother during childbirth, and data was collected from a limited number of hospitals, taking the sample became closer to 5 people per item i.e. 110 people surveyed, which was another limitation of the study. Moreover, the sample contained a predominantly highly educated (92 % university educated) proportion of society, meaning it is not representative of the general population. Items 13 and 14 were removed because they did not provide the desired homogeneity in the Turkish version. These items are related to clinical differences and are a weakness of this version of the questionnaire. Also, demographic information wasn't collected concerning the mothers. It should also be noted that current population migrations are a factor to be considered in any new study of health systems in developing countries. The study's strength lies in the reliability of the tool that measures the childbirth experience for the first time for fathers in Türkiye.

## 7 Conclusion

Results of the present study indicated that the Turkish version of the FTFQ was culturally sufficient and reliable. Moreover, it was found to be a valid questionnaire for assessing the first time delivery-related experiences of new fathers. The use of this questionnaire will contribute to the performance of different studies that will assess the delivery-related experiences of first-time fathers and reveal the paternal contribution to the delivery process. It can facilitate further actions to improve paternal satisfaction and the fathers’ behaviour as a labour companion.

## Financing of the study

No financial support was received in this study.

## Data availability statement

Data will be made available on request to the first author.

## CRediT authorship contribution statement

**Ayça Demir Yildirim:** Writing – original draft, Visualization, Validation, Resources, Project administration, Methodology, Investigation, Formal analysis, Data curation, Conceptualization. **Tuğba Yilmaz Esencan:** Writing – review & editing, Writing – original draft, Visualization, Validation, Supervision, Software, Resources, Methodology, Investigation, Formal analysis, Conceptualization. **Asa Premberg:** Writing – review & editing, Validation, Supervision, Resources, Methodology, Conceptualization. **Nevin Hotun Şahin:** Writing – review & editing, Validation, Supervision, Software, Resources, Methodology, Formal analysis, Conceptualization.

## Declaration of competing interest

The authors declare that they have no known competing financial interests or personal relationships that could have appeared to influence the work reported in this paper.
